# Efficacy of tripterygium glycosides for diabetic nephropathy: a meta-analysis of randomized controlled trials

**DOI:** 10.1186/s12882-021-02487-8

**Published:** 2021-09-07

**Authors:** Hua-Bin Guo, Jia-Qing Peng, Xuan Wang, Ke-Kai Zhang, Guang-Zhi Zhong, Wei-Hong Chen, Gui-Xin Shi

**Affiliations:** 1 Yangtze University Health Science Center, 434023 Jingzhou, China; 2 Department of Nephrology, Jingzhou Central Hospital, 434020 Jingzhou, China; 3 Department of Nephrology, Ankang Hospital of Traditional Chinese Medicine, 725000 Ankang, China; 4 Hanyin County Hospital of Traditional Chinese Medicine, 725100 Hanyin, China

**Keywords:** Tripterygium glycosides, Diabetic nephropathy, randomized controlled trials, Meta-analysis

## Abstract

**Backgrounds:**

Diabetic nephropathy (DN) is one of the most important clinical complications of diabetes mellitus (DM) and is the most common cause of end-stage renal disease. Currently, there is no highly effective medicine that can prevent, halt, or reverse the progressive course of DN. Initial clinical data showed that Tripterygium glycosides (TGs), a traditional Chinese medicine, can decrease proteinuria in patients with DN.

**Objectives:**

The objective of the present study is to investigate the efficacy and safety of TGs for the treatment of DN through meta-analysis of randomized controlled trials (RCTs).

**Methods:**

All RCTs of TGs for DN were collected from The China National Knowledge Infrastructure (CNKI), PubMed, Web of Science, Wanfang Data, Chinese Biomedical Literature Database (CBM), China Science and Technology Journal Database (VIP) by setting the study inclusion and elimination standards. Two reviewers evaluated the quality of the trials and extracted the data independently. RevMan 5.4 software was used for meta-analyses. The primary outcome was a change in 24-hours urinary total protein (24 h TUP).

**Results:**

26 RCTs with 1824 participants were identified. Studies were assessed using the Cochrane risk of bias tool. The overall effects showed that TGs was compared with the controls, TGs showed significant effects in reducing 24 h TUP [WMD = -0.84, 95 % *CI* (-1.09, -0.59)], elevating serum albumin [WMD = 2.88, 95 % *CI* (1.87, 3.90)], and the total efficiency [*OR* = 4.08, 95 % *CI* (2.37, 7.04)]. This effect was consistent across the subgroups of period of intervention.

**Conclusions:**

The present research showed that TGs was significantly associated with improvement of renal function in patients with DN. TGs offers a novel approach to the treatment of DN, more high-quality RCTs are needed for a better understanding of the role of TGs in DN therapy.

**Supplementary Information:**

The online version contains supplementary material available at 10.1186/s12882-021-02487-8.

## Background

Diabetic nephropathy (DN) is one of the most important clinical complications of diabetes mellitus (DM), recently also named diabetic kidney disease (DKD), and is the most common cause of chronic kidney diseases [1]. DN is the leading cause of chronic kidney disease in patients starting renal replacement therapy. With the prevalence of diabetes, the incidence of DN is growing rapidly, which imposes a huge economic burden on our society [2, 3]. pathological changes of clinical DN patients are characterized by glomerular mesangial matrix expansion, glomerular basement membrane (GBM) thickening, and formation of glomerular nodular sclerosis [4, 5]. The clinical manifestations of DN include hypertension, massive proteinuria, edema, and progressive decrease in renal function [6]. The precise mechanisms of DN pathogenesis are yet to be fully elucidated. The studies were from more recent years show that inflammation played a vital role in the pathogenesis of DN [7]. It is believed that persistent hyperglycemia plays a positive role in the pathogenesis of DN, but simply controlling blood glucose doesn’t stop the progression of DN symptoms [8]. How to select new drugs for diabetic nephropathy from the perspective of anti-inflammatory and immunosuppressive is a subject for kidney disease workers.

Tripterygium wilfordii is a traditional Chinese medicine, its extract Tripterygium glycosides (TGs) belong to non-steroidal immunosuppressants, can effectively inhibit cellular and humoral immunity [9, 10]. In 1977, Chinese scholars first reported its role in reducing proteinuria in glomerulonephritis, and TGs began to be used in the clinical treatment of renal diseases. TGs have been extensively used in China for the treatment of autoimmune diseases, such as rheumatoid arthritis, primary glomerulonephritis, and immune-related nephritis [11–13]. At present, some clinical studies apply TGs in the treatment of DN, It is suggested that TGs can antagonize the anti-inflammatory effect on the pathogenesis of DN and thus protect renal function [14]. However, the results of these studies are inconsistent, and there are fewer detailed reports on the side effects of TGs. Therefore, this study systematically evaluated the efficacy and safety of TGs in the treatment of DN on the basis of existing RCTs, Aimed at providing evidence-based medical evidence for the clinical application of TGs in the treatment of DN.

## Methods

### Search strategy

We searched RCTs via PubMed, Medline, Embase,China National Knowledge Infrastructure (CNKI), Web of Science, Wanfang Data, Chinese Biomedical Literature Database (CBM), China Science and Technology Journal Database (VIP) up to June 2020. The following keywords and Medical Subject Heading (MeSH) were searched: “diabetic kidney disease” or “diabetic nephropathies” or “diabetic glomerulosclerosis”, and “Tripterygium wilfordii Hook” or “tripterygium glycosides”. To collect an adequate number of trials, the reference lists of pertinent publications were also retrieved to identify additional studies.

### Inclusion and exclusion criteria

Included criteria: Trials were considered to be eligible for inclusion if they met all of the following criteria: (1) Research type: studies based on RCTs. (2) Research subjects: the patients of the original studies have been clinically diagnosed with DN. (3) Interventions: The control group was treated with conventional treatment and the experimental group was added with TGs on the basis of the control group. Other measures were consistent with the two groups. Control group: participants received intervention of intensive blood glucose control, intensive blood pressure treatment and renin-angiotensin system blockade, Diet intervention. Without sodium glucose co-transporter (SGLT2) inhibitors adopted for the prevention of DN. (4) Outcome measures: 24 h urinary total protein (24 h UTP), serum creatinine (SCr), blood urea nitrogen (BUN), creatinine clearance (Ccr), albumin (Alb), alanine aminotransferase (ALT), white blood cells (WBC). Elevated ALT is a serological manifestation of liver damage, and reduced WBC is a serological manifestation of myelosuppression. Liver function damage and myelosuppression are considered to be major adverse reactions. Total efficiency was defined as obvious effect plus effective according to guidelines for clinical research of new Chinese medicine drugs [15, 16].

Exclusion criteria: (1) Non-randomized controlled trials. (2) Participants had comorbidities. (3) Other Chinese medicines being used in the control group or the experimental group. (4) The publications lacked original data for the meta-analysis and review articles. Pieces of literature that lack rigorous experimental design, inappropriate methodological or statistical analysis, or lack of relevant outcome measurements will be excluded.

### Data extraction

**Fig. 1:**
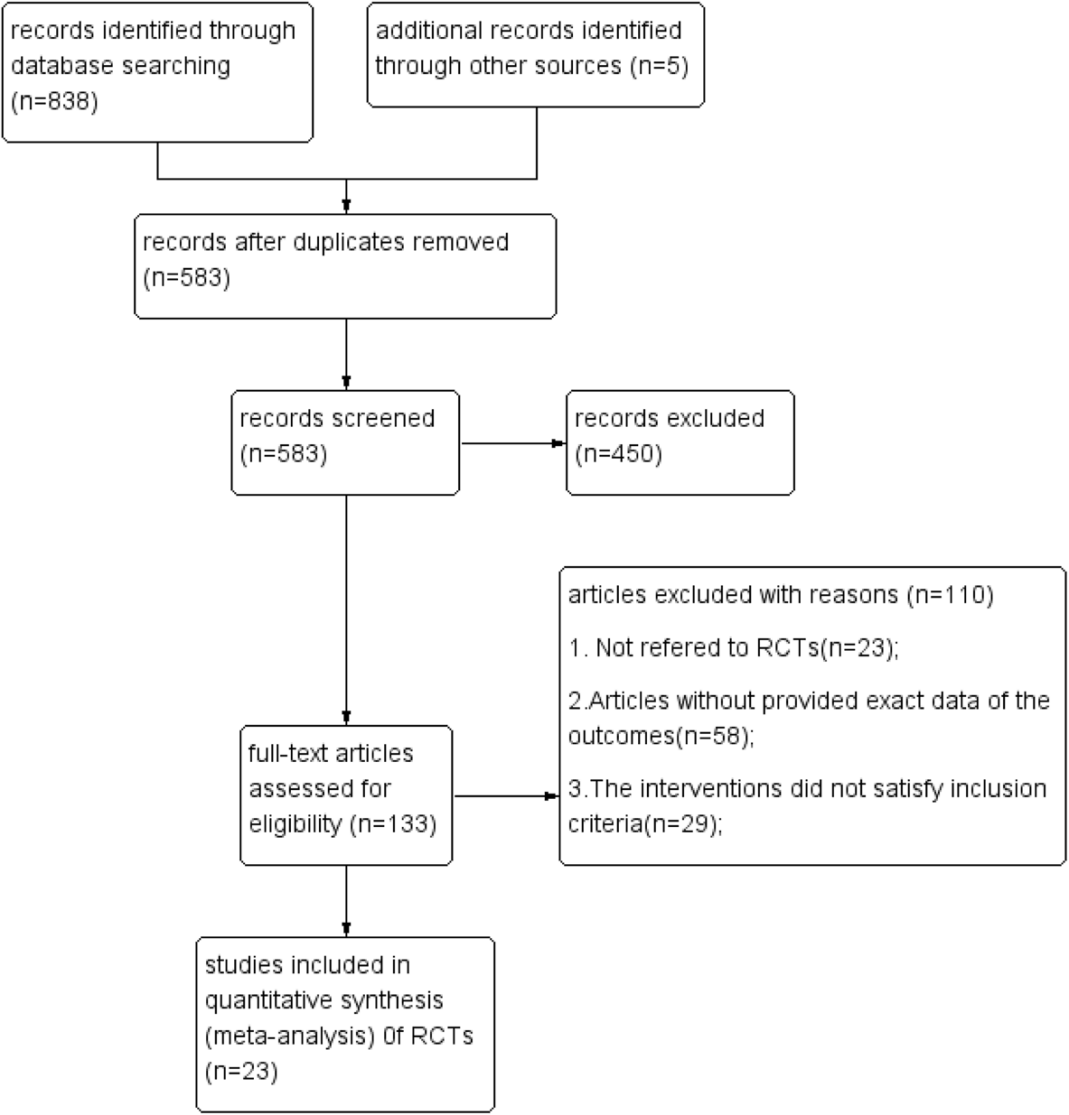
Flow diagram of study identification process

Two examiners (Gui-Xin Shi, Xuan Wang) selected the articles and extracted the relevant data independently, and contradictions were resolved by consensus or were judged by another reviewer (Ke-Kai Zhang). The extracted data of included articles included study design, randomization, diagnostic criteria, the first author’s name, publication year, sample size (treatment group, control group), intervention and dosing regimen (treatment group, control group), duration of treatment, major outcome. The process of study selection was shown in Fig. 1.

### Quality assessment

**Fig. 2:**
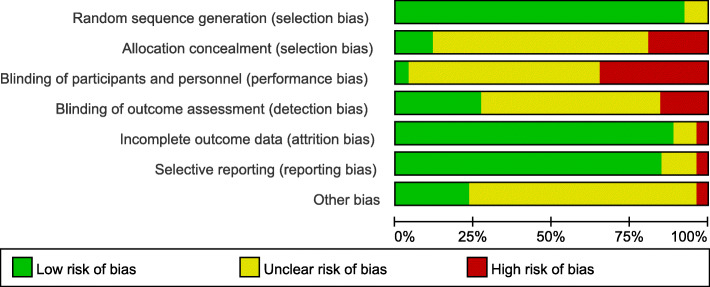
Risk of bias graph: Each risk of bias item was included for each study

We evaluated the quality of studies using the Review Manager (vision 5.4.1) risk-of-bias tool. The tool includes the following 7 sections: random sequence generation (selection bias), allocation concealment (selection bias), blinding of participants and personnel (performance bias), blinding of outcome assessment (detection bias), incomplete outcome data (attrition bias), selective reporting (reporting bias) and other biases. ( Fig. 2 and Supplemental Figure 1)

### Statistical analysis

We used the Review Manager (version 5.4.1) and STATA (version 12.0, Stata SE) software for statistical analysis. Odds ratio (OR) were used to assess dichotomous data, and standardized mean difference (SMD) and mean difference (MD) were used to assess continuous data, and 95 % confidence intervals (CI) were calculated for continuous data. We used I^2^ statistic to assess the heterogeneity of included RCTs. If there was homogeneity (*P* > 0.1, *I*^*2*^ < 50 %) in the results, we used a fixed-effect model. If I^2^ was higher than 50 %, sensitivity analysis and subgroup analysis performed to explore possible sources of heterogeneity. If heterogeneity remains higher than 50 %, we used a random-effect model to analyze data. Quantitative methods such as Begg’s and Egger’s tests will be used to assess publication bias.

## Results

### Study characteristics

We selected and read 26 RCTs in full to use for the meta-analysis [17–42]. A total of 26 trials including 939 DN Patients receiving TGs and 885 controls that met our inclusion criteria were included in the present study. It should be noted that the guideline-recommended conventional treatments for DN, such as intensive glucose and blood pressure management and dietary interventions, were the same in both groups in all included studies, and therefore these conventional treatments are not mentioned separately below. Enrolled participants were diabetic patients with persistent albuminuria or proteinuria, but differed in baseline renal function and were not treated with SGLT2 inhibitors. All the studies were conducted in China. The characteristics of included studies are summarized in Supplemental Table 1.

### 24-hours urinary total protein (24 h UTP)

Difference in 24-hours urinary total protein (24 h UTP) were found between the experimental group and control group of twenty-four trials.^[17–21,23−27,29−42]^ The results were included in RevMan5.4.1 software. Considering the significant heterogeneity (*P* < 0.05, *I*^*2*^ = 85 %), Therefore, the random-effect model was adopted. The pooled results indicated that the TGs group is more effective at reducing 24 h UTP [WMD = -0.84, 95 % CI (-1.09, -0.59)].

**Fig. 3:**
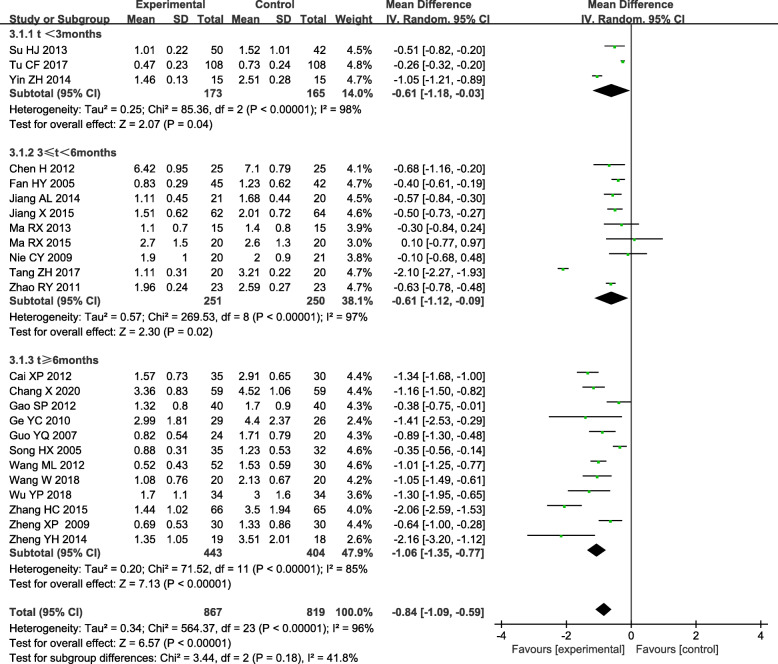
Subgroup analysis of TGs in the treatment of DN based on the duration of treatment, outcome: 24 h TUP

The subgroups were divided into t < 3 months, t < 6 months and t > 6 months of TGs compared to control treatment (Fig. 3). There was still obvious heterogeneity within each subgroup [t < 3 months: (*I*^*2*^ = 98 %, *P* < 0.05); 3 < t < 6 months: (*I*^*2*^ = 97 %, *P* < 0.05); t > 6 months: (*I*^*2*^ = 85 %, *P* < 0.05)]. Subgroup analysis also showed that there was a statistically significant difference in 24 h UTP between the experimental group and control group, experiment group was superior to the control group in reducing the 24 h UTP [t < 3 months: WMD = -0.61, 95 % CI(-1.18, -0.03); 3 < t < 6 months: WMD = -0.61, 95 % CI (-1.12, -0.09); t > 6 months: WMD = -1.06, 95 % CI (-1.35, -0.77)]. The pooled results showed that when the duration of treatment was t ≥ 3 months, the intervention group was more meaningful in reducing 24 h UTP compared to the control group.

**Fig. 4:**
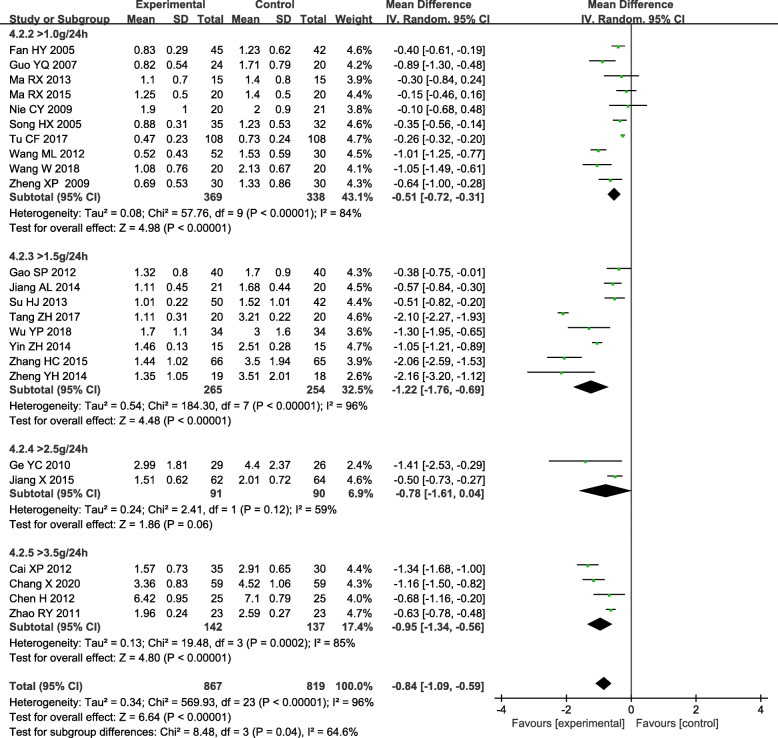
Subgroup analysis of TGs in the treatment of DN based on the 24 h UTP baseline of inclusion criteria, outcome: 24 h UTP

The subgroup based on 24 h UTP baseline of inclusion criteria analysis showed there was still obvious heterogeneity within each subgroup [> 1.0 g/24 h: (*P* < 0.05, *I*^*2*^ = 84 %); >1.5 g/24 h: (*P* < 0.05, *I*^*2*^ = 96 %); >2.5 g/24 h: (*P* < 0.05, *I*^*2*^ = 59 %); >3.5 g/24 h: (*P* < 0.05, *I*^*2*^ = 85 %)]. Other subgroups indicated the combined group was superior to the control group in reducing the 24 h UTP [> 1.0 g/24 h: WMD= -0.51, 95 % CI (-0.72, -0.31); >1.5 g/24 h: WMD = -1.22, 95 % CI (-1.76, -0.69); >3.5 g/24 h: WMD = -0.95, 95 % CI (-1.34, -0.56)] (Fig. 4).

### Serum albumin (Alb)

**Fig. 5:**
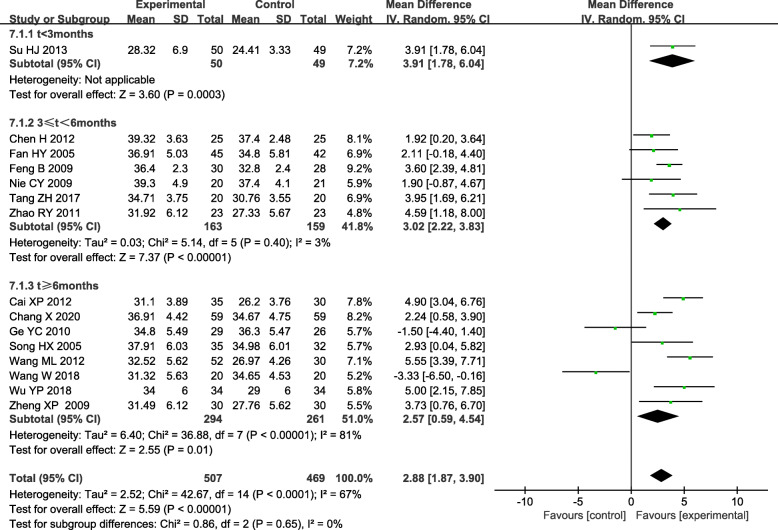
Subgroup analysis of TGs in the treatment of DN based on the course of treatment, outcome: Alb

Persistent albuminuria is a hallmark of DN, and albuminuria is a strong predictor of serum albumin (Alb) levels [43]. Additionally, patients with DN commonly have comorbid conditions, fluid overload, protein-energy wasting, and inflammation, all of which are known to affect serum albumin concentrations [44], and serum albumin is predominantly low in patients with DN [45]. Seventeen trials reported a difference in Alb between the experimental and control group [17–22, 24, 27, 30–37]. Two trials with Alb over 40 g/L after treatment were excluded [29, 32]. Statistical heterogeneity analysis indicated significant heterogeneity across the studies(*I*^*2*^ = 67.0 %, *P* < 0.05). Therefore, Results conducted by random-effect model showed the experimental group was superior to the control group in elevating Alb [WMD = 2.88, 95 % CI (1.87, 3.90)]. Subgroup analysis based on the course of treatment. All subgroups indicated the experimental group was superior to the control group in elevating Alb [3 ≤ t < 6months: WMD = 3.02, 95 % CI (2.22, 3.83); t > 6 months: WMD = 2.57, 95 % CI (0.59, 4.54)]. (Fig. 5).

### 3.4. Serum creatinine (SCr)

**Fig. 6:**
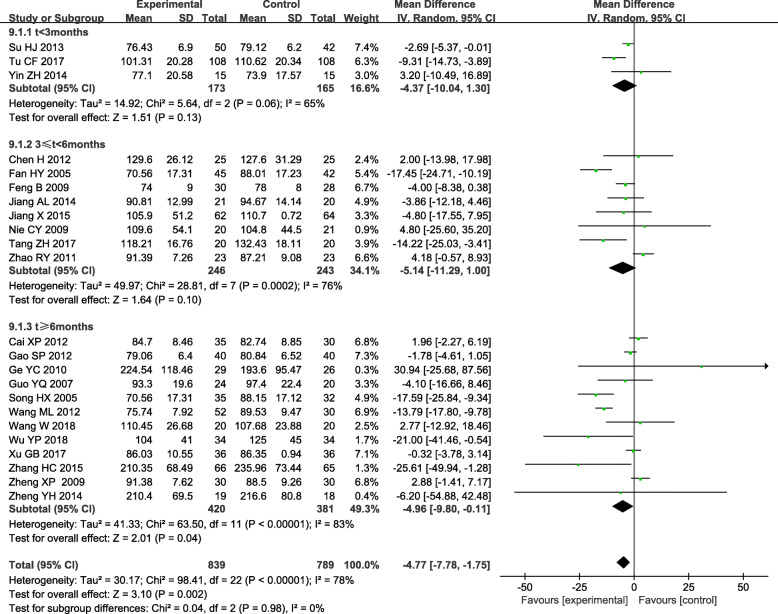
Subgroup analysis of TGs in the treatment of DN based on the course of treatment, outcome: SCr

Twenty-three trials demonstrated a difference in Serum creatinine (SCr) between the experiment and control group.^[17–37,41−42]^ Significant heterogeneity (*P* < 0.05, *I*^*2*^ = 78 %)was identified and analyzed data by a random-effect model. Meta-analysis showed that TGs could improve SCr better than control group [WMD= -4.77; 95 % CI (-4.78, -1.75)] in DN patients. Subgroup analysis based on the course of treatment showed There was still significant heterogeneity within each subgroup [t < 3months: (*P* = 0.06, *I*^*2*^ = 65.0 %); 3 < t < 6 months: (*P* < 0.05, *I*^*2*^ = 76.0 %); t > 6 months: (*P* < 0.05, *I*^*2*^ = 83.0 %)]. Subgroup analysis showed there was no difference in SCr between the experimental group and control group if treatment lasted less than 3 months and 6 months [t < 3 months: WMD = -4.37, 95 % CI (-10.04, 1.30);3 < t < 6 months: WMD= -5.14, 95 % CI (-11.29, 1.00)]. But there was a significant difference in SCr between the experimental group and control group if the course of treatment more than 6 months (WMD= -4.96, 95 % CI (-9.80, -0.11). (Fig. 6).

### Blood urea nitrogen (BUN)

**Fig. 7:**
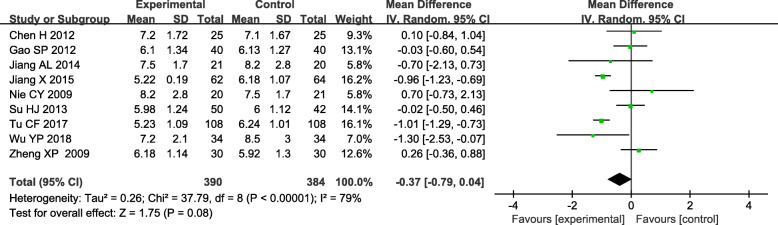
Forest plots of TGs in the treatment of DN, outcome: BUN

Blood urea nitrogen (BUN) also is an indicator of kidney function, often used to reflect the change of kidney function of DN patients. Nine studies reported a difference in BUN between the experimental and the control group. ^[19–20,25−27,30, 33,37,41]^ The significant heterogeneity was found across these studies (*P* < 0.05, *I*^*2*^ = 79 %). Therefore, the data were pooled by a random effect model. The result indicated that there was no significant difference in reducing BUN between TGs and routine treatment in DN patients [WMD= -0.37; 95 % CI (-0.79, 0.04); *P* = 0.08)]. (Fig. 7).

### 3.6. Creatinine clearance (CCr)

**Fig. 8:**
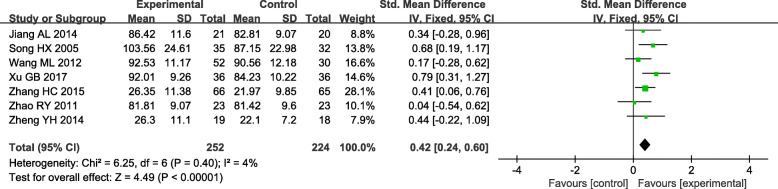
Forest plots of TGs in the treatment of DN, outcome: CCr

Creatinine clearance (CCr) as the main indicator of kidney function was reduced in DN Patients. Seven trials reported a difference in CCr between the experiment and control group.^[21,28–29,32−33,35,42]^ Statistical heterogeneity analysis indicated slow heterogeneity across the studies(*I*^*2*^ = 4.0 %, *P* = 0.40). Therefore, a fixed-effect model was used. The result showed that the improvement to CCr by TGs treatment was better than conventional treatment [(SMD = 0.42; 95 % CI (0.24, 0.60)]. (Fig. 8).

### Adverse reactions

**Fig. 9:**
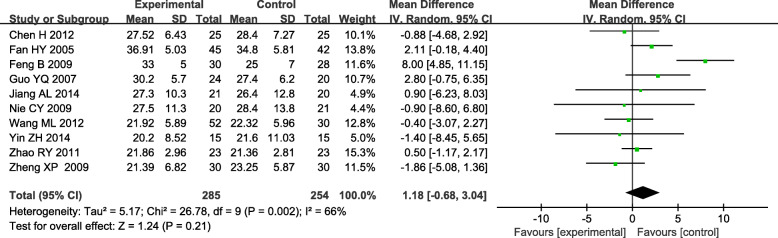
Forest plots of TGs in the treatment of DN, outcome: ALT

Alanine aminotransferase (ALT) is a measurement of liver damage in TGs. The effect on ALT was mentioned in ten studies and significant heterogeneity was found across these studies (*P* < 0.05, *I*^*2*^ = 66 %) [19, 20, 22, 23, 32, 33, 35, 36]. Therefore, the data were pooled by a random-effect model. The result indicated that the TGs did not cause ALT elevation compared to the control because there was no significant difference between the experimental and the control group [WMD = 1.18, 95 % CI (-0.68, 3.04), *P* = 0.21]. (Fig. 9).

**Fig. 10:**
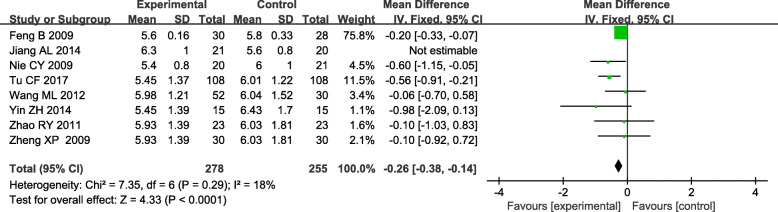
Forest plots of TGs in the treatment of DN, outcome: WBC

The white blood cells (WBC) were mentioned in eight studies and significant heterogeneity was found across these studies (*P* = 0.01, *I*^*2*^ = 62 %) [19, 22, 25, 32, 33, 35–37]. We conducted a sensitivity analysis by deleting a study to eliminate heterogeneity (*P* = 0.29, I^2^ = 18 %) [29]. Therefore, a fixed-effect model was used. The result showed TGs could make reduction in WBC than routine treatment [(WMD= -0.26; 95 % CI (-0.38, -0.14)] ( Fig. 10).

### Total efficacy

**Fig. 11:**
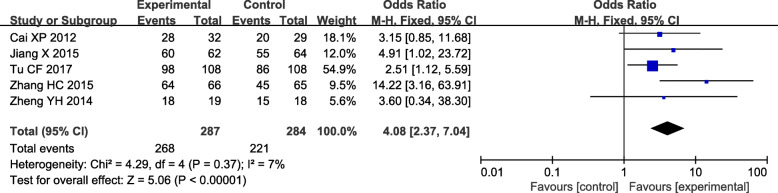
Forest plots of the total efficiency of TGs in the treatment of DN

Total efficiency was all evaluated in interventions and controls in five trials [17, 25, 26, 29, 42]. There was a slow heterogeneity in the total efficiency (*I*^*2*^ = 7.0 %, *P* = 0.37). Thus, the experimental odds ratio (OR) was pooled by a fixed-effect model. The total efficiency of the experimental group was obviously higher than the control [OR = 4.08, 95 % CI (2.37, 7.04)] (Fig. 11).

### Sensitivity analysis and publication bias

Sensitivity analysis can assess the stability and reliability of the conclusions of the meta-analysis. Sensitivity analysis with 24 h UTP and SCr in Stata 12.0 software. The results illustrated that the meta-analysis about 24 h UTP and SCr had low sensitivity and high stability in the analysis of patients with DN. (Supplemental Figure 2 and Figure 3). Qualitative analysis of the funnel plot and graph symmetry was used to assess publication bias. The publication bias of 24 h UTP and SCr for included RCTs was evaluated by comparing the symmetry of the funnel plot. (Supplemental Figure 4 and Figure 5). Begg’s and Egger’s test with 24 h UTP and SCr in Stata12.0 to further confirm whether the publication bias for 24 h UTP and SCr. The result to 24 h UTP, Egger (t = − 1.95, *P* > 0.05) and Begg test (Pr >|Z| = 0.47). The result to SCr, Egger (t = − 0.92, *P* > 0.05) and Begg test (Pr >|Z| = 1.00). Which indicated that there was no evidence of substantial publication bias. However, it is needed to highlight that the quality of RCTs included restricted this application.

## Discussion

DN is a progressive kidney disease caused by damage to the capillaries in the kidneys’ glomeruli [46]. DN is a leading cause of end-stage renal disease worldwide [47]. The pathogenesis of DN includes many factors such as hemodynamics, Inflammation, immune reactions, oxidative stress, podocytes injury [47, 48]. There are still no effective treatments for DN, the purposes of treatment for DN were to decrease the progression of kidney damage and control proteinuria, hypoproteinemia, and other associated complications.

The known risk factors of DN are hypertension, hyperglycemia, smoking, and dyslipidemia, which should be treated vigorously as the basis of DN treatment. The prevention of diabetic nephropathy adopts the therapeutic principles of strengthening blood glucose, controlling blood pressure, and blocking the renin-angiotensin system. The major treatment modality of diabetic nephropathy was angiotensin-converting enzyme inhibitor/angiotensin receptor blockers (ARB/ACEI) [49]. Treatment of ARB/ACEI can generally reduce proteinuria and alleviate the progression of kidney injury.

Several recent studies have shown that SGLT2 inhibitors slow the year-to-year loss of kidney function and might also reduce the risk of the renal composite outcome. However, the efficacy of the currently available ARBs or SGLT2 for the treatment of DN is insufficient. Furthermore, other methods including blood pressure control, blood sugar management, and low-protein diets have been used to treat DN and partially delay its progression. Traditional Chinese Medicine (TCM) has a long history of treating DN in China, and over long empirical practice, practitioners have gained a deeper understanding of the development of DN and proposed the evolution of its pathogenesis. In recent years, the potential and positive effects of TCM have increasingly attracted public interest in the treatment of DN. Some traditional Chinese herbs have been proven to be very effective in the treatment of kidney diseases, such as Astragalus and TGs [12, 50]. In China, TGs has been widely used for many years to treat primary kidney diseases and immune-related nephritis [51, 52]. In the last few years, several prospective clinical studies have discussed the clinical efficacy of TGs in the treatment of DN [53, 54]. Most of the studies showed that TGs can improve clinical efficacy, decrease the 24 h urinary protein, and serum creatinine. In DN, TGs not only improves proteinuria, but also alleviates kidney pathological changes and reduced the levels of inflammation in the kidney via p38 mapk pathway [55, 56].

The mechanisms by which TGs treat DN have not been clearly clarified. The immunologic and inflammatory mechanisms may play important roles in the development and progression of DN [57], The protective effects of TGs are in part due to its immunosuppressive, anti-inflammatory, anti-oxidative stress effects. TGs may have a possible mechanism to reduce proteinuria by downregulation of the expression of oxidative carbonyl protein (OCP) in the renal cortex of DN [2].

In our present meta-analysis, 26 clinical studies were included, including 1824 subjects (939 in the experiment group and 885 in the control group). we found that the TGs group showed significant effects in reducing 24 h UTP (*P* < 0.05), elevating Alb (*P* < 0.05), reducing Scr (*P* < 0.05), increasing Ccr (*P* < 0.05), and increasing the total efficiency when compared to the control group, which is the same as others meta-analysis [15, 58, 59]. However, TGs treatment had no obvious advantage in improving BUN (*P* > 0.05) and increasing ALT (*P* > 0.05), The adverse effects of bone marrow suppression are obvious in the TGs group, there was a significant effect on reducing WBC (*P* < 0.05) when compared to the control group. The present evidence indicate that TGs can improve clinical efficacy, reduce the 24 h urinary protein, and serum creatinine as compared with conventional treatment. Thus, We can speculate that TGs can repair the renal function to some extent in DN patients compared with controls, but the pooled results show that the associated toxicity of bone marrow suppression with TGs is significantly higher than that of the control group. In conclusion, this study may provide information on a safe and effective way to reduce urinary protein and delay the progression of kidney disease in patients with diabetic nephropathy.

There were several limitations in this meta-analysis that should be taken into consideration when interpreting the results. (1) Only Chinese and English studies were included in this meta-analysis, subjects in this study focused on the Chinese population and caution should be exercised when applying the study results to patients of other nationalities; (2) The general quality of the included studies were poor, most studies did not describe allocation concealment and blinding, These are most likely to cause selection bias, attrition bias, and reporting bias; (3) The diagnostic criteria for DN patients are not uniform, which may lead to different stages of DN for patients included; (4) significant statistical heterogeneity still existed across the included studies and should be further explored.

## Supplementary Information

**Additional file 1:**

## Data Availability

Data supporting the results are reported in this article and additional information is available. In addition, relevant materials used in the study are available from the corresponding authorson reasonable request.

## Abbreviations

DN: Diabetic nephropathy
TGs: Tripterygium glycosides
RCTs: Randomized controlled trials
CNKI: China National Knowledge Infrastructure
CBM: Chinese Biomedical Literature Database
24 h TUP: 24-hours urinary total protein
DM: Diabetes mellitus
GBM: Glomerular basement membrane
MeSH: Medical Subject Heading
SCr: Serum creatinine
BUN: Blood urea nitrogen
Ccr: Creatinine clearance
Alb: Albumin
ALT: Alanine aminotransferase
WBC: White blood cells
