# Supplementary material for: Efficacy of tripterygium glycosides for diabetic nephropathy: a meta-analysis of randomized controlled trials
Source: BMC Nephrol. 2021 Sep 7;22:304. doi: 10.1186/s12882-021-02487-8 (PMC8425142; doi:10.1186/s12882-021-02487-8)

**Supplemental Figure 1.**Risk of bias summary: Each risk of bias item was included for each study.

**
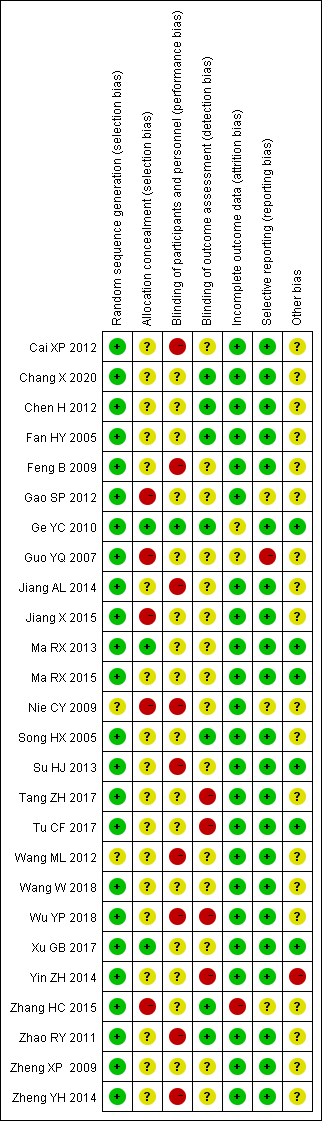
**

**Supplemental Figure 2.** Sensitivity analysis shows the meta-analysis has low sensitivity and high stability for analyzing 24h TUP of patients with DN after the treatment of TGs.


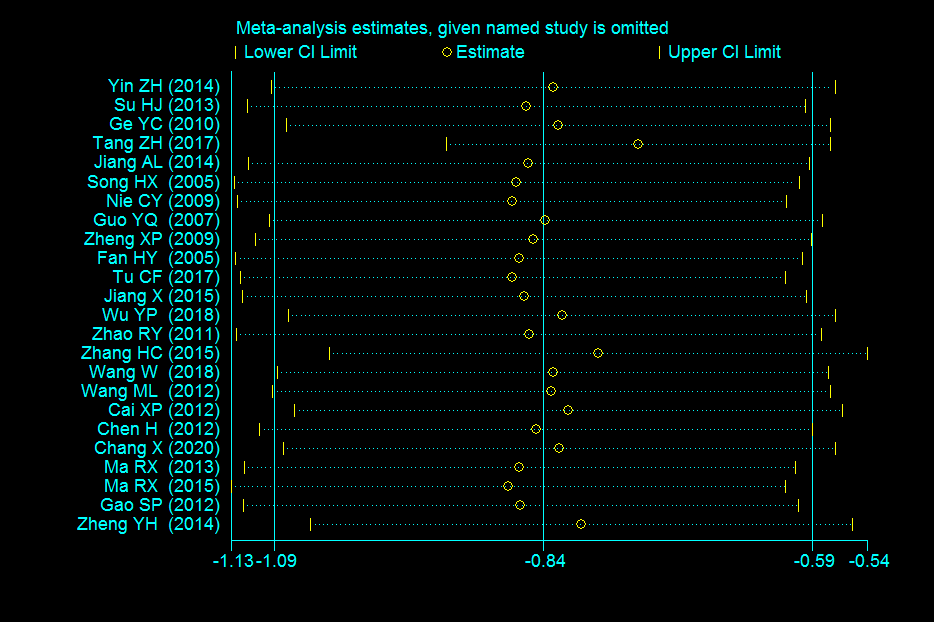


**Supplemental Figure 3.** Sensitivity analysis shows the meta-analysis has low sensitivity and high stability for analyzing Scr of patients with DN after the treatment of TGs.


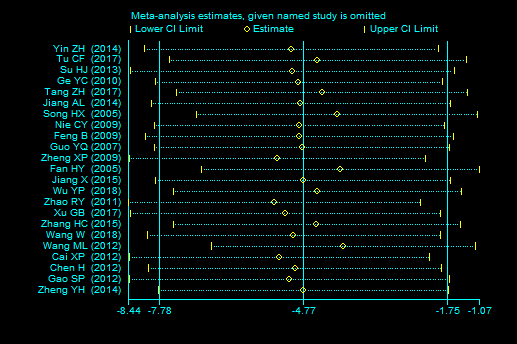


**SupplementalFigure4.**The funnel plot of 24h TUP for included RCTs.


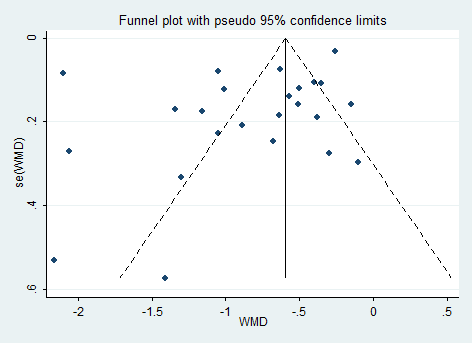


**SupplementalFigure5.**The funnel plot ofScr for included RCTs.


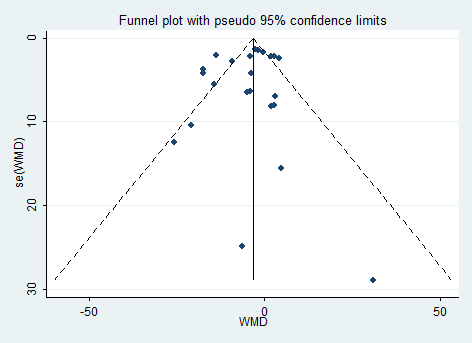


**Supplemental Table 1.** The characteristics of included studies

| Study, Year | Sample size (T/C) | Control | Experimental | Course (month) | Outcome | UTP baseline of inclusion criteria |
| --- | --- | --- | --- | --- | --- | --- |
| Cai XP 2012^[17]^ | 35/30 | Valsartan 40-80mg/d | TGs 60mg/d + Control group treatment | 6m | 1,2,3,8 | >3.5 g/24h |
| Wang W 2018^[18]^ | 20/20 | Routine treatment | TGs 60mg/d + Control group treatment | 6m | 1,2,3 | >1.0 g/24h |
| Nie CY 2009^[19]^ | 21/20 | Routine treatment | TGs 40mg/d + Control group treatment | 3m | 1,2,3,4,6,7 | >1.0 g/24h |
| Chen H 2012^[20]^ | 25/25 | Irbesartan 150mg/d | TGs 40mg/d + Control group treatment | 3m | 1,2,3,4,7 | ≥1.0 g/24h |
| Song HX 2005^[21]^ | 35/32 | benazepril 5～ 20 mg/d | TGs 1~2mg/(kg.d) + Control group treatment | 6m | 1,2,3,5 | ≥1.0g/24h |
| Feng B 2009^[22]^ | 30/28 | Irbesartan 150m/d | TG 60mg/d + Control group treatment | 3m | 2,3,4,6 | ≥1.0g/24h |
| Guo YQ 2007^[23]^ | 24/20 | Routine treatment | 1~2months:TGs 60mg/d; 3-6months: TGs 40mg/d + Control group treatment | 6m | 1,3,4 | ≥1.0g/24h |
| Fan HY 2005^[24]^ | 45/42 | Routine treatment | TGs 1~2mg/(kg.d) + Control group treatment | 3m | 1,2,3 | ≥1.0 g/24 h |
| Tu CF 2017^[25]^ | 108/108 | Telmisartan 40mg/d | TGs 1.5mg/( kg.d) + Control group treatment | 1m | 1,3,6,7,8 | ≥1.0g/24h |
| Jiang X 2015^[26]^ | 62/64 | Telmisartan 80 mg/ d | TGs 60mg/d + Control group treatment | 3m | 1,3,7,8 | ≥2.5g/24h |
| Wu YP 2018^[27]^ | 34/34 | Losartan 80～160 mg/d | 1~2months: TGs 60mg/d; 3-6months: TGs 30mg/d + Control group treatment | 6m | 1,2,3,7 | ≥1.0g/24h |
| Xu GB 2017^[28]^ | 36/36 | Routine treatment | 1 month: TGs 60mg/d; 2~6months 30 mg/d + Control group treatment | 6m | 3,5 | 30 ~300 mg/24h |
| Zhang HC 2015^[29]^ | 66/65 | Losartan 80mg/d | TGs 60mg/d + Control group treatment | 12m | 1,3,5,8 | ≥0.5g/24 h |
| Su HJ 2013^[30]^ | 50/42 | Routine treatment | TGs 60mg/d + Control group treatment | 2.5m | 1,2,3,7 | ≥1.5g/24 h |
| Ge YC 2010^[31]^ | 34/31 | Losartan 160mg/d | 1~2 months:TG 120mg/d; 3~4months 60mg/d + Control group treatment | 6m | 1,2,3 | ≥2.5g/24 h |
| Wang ML 2012^[32]^ | 52/30 | Losartan 160mg/d | 1~2 months:TG 60mg/d; 3~4months 20mg/d + Control group treatment | 6m | 1,2,3,5,4,6 | ≥1.0 g/24 h |
| Jiang AL 2014^[33]^ | 21/20 | Routine treatment | TGs 1.5mg/( kg.d) + Control group treatment | 3m | 1,2,3,5,4,6,7 | ≥1.0 g/24 h |
| Tang ZH 2017^[34]^ | 20/20 | Routine treatment | TGs 60mg/d + Control group treatment | 3m | 1,2,3 | ＞0.9 g/24 h |
| Zhao RY 2011^[35]^ | 23/23 | Losartan 160mg/d | TGs 60mg/d + Control group treatment | 3m | 1,2,3,5,4,6 | ≥1.5g/24 h |
| Yin ZH 2014^[36]^ | 15/13 | Routine treatment | TGs 60mg/d + Control group treatment | 2m | 1,2,3,4,6 | ≥2.0g/24 h |
| Zheng XP 2009^[37]^ | 30/30 | Routine treatment | 1~2 months: 1mg/(kg·d); 3~6months 0.5 mg/(kg· d) + Control group treatment | 6m | 1,2,3,6,7 | ≥0.9g/24 h |
| Ma RX 2013^[38]^ | 15/15 | Irbesartan 150m/d | TGs 1～2 mg/(kg·d) + Control group treatment | 3m | 1 | ≥1.0 g/24 h |
| Chang X 2020^[39]^ | 59/59 | Losartan 160mg/d | TGs 60 mg/d + Control group treatment | 6m | 1,2 | ≥3.0 g/24 h |
| Ma RX 2015^[40]^ | 20/20 | Irbesartan 150m/d | TGs 1～2 mg/(kg·d) + Control group treatment | 3m | 1, | ≥1.0 g/24 h |
| Gao SP 2012^[41]^ | 40/40 | Routine treatment | TGs 60 mg/d + Control group treatment | 6m | 1,3,7 | ≥1.5 g/24 h |
| Zheng YH 2014^[42]^ | 19/18 | Routine treatment | TGs 60 mg/d + Control group treatment | 12m | 1,3,5,8 | ≥0.5 g/24 h |

Abbreviations: TGs, Tripterygiumglycosides; UTP, urinary total protein; T/C. trials/control Outcomes: (1) 24-hour urinary protein (g/24h); (2) Serum albumin(g/L); (3) Serum creatinine (μmol/L); (4)alanineaminotransferase(mmol/L);(5)creatinine clearance(ml/min); (6)white blood cells(10^9^/L), (7)blood urea nitrogen(mmol/L),and (8) total efficacy

**References:**

[17] Cai XP (2012) ARB combined with Tripterygium glycosides in the treatment of diabetic nephropathy. Journal of Clinical Medicine in Practice 16: 112-114.

[18] Wang W (2018) Different Doses of Tripterygium Glycosides in the Treatment of Diabetic Nephropathy: Effects on Blood Lipids. Kidney Blood Pressure Research. 43:931-937.

[19] Nie CY, Chen LM, Chang BC, et al (2009) The effect of tripterygium wilfordii on proteinuria in patients with diabetic nephropathy. Chinese Journal of Practical Internal Medicine 29:517-519.

[20] Chen H, Zhuang LP, Liu JF, et al (2012) Effect of Irbesartan and Triptolide com bination on the level of urine protein in patients with diabetic nephropathy at high altitude area. Clinical Medicine of China11:1149-1151.

[21] Song HX, Gong J, Chen W et al (2005) Effects of Tripterygium glycosides on urinary monocyte chemotaxis protein -1 in diabetic nephropathy patients. Chinese Journal of Integrated Traditional and Western Medicine2:416-418.

[22] Feng B, Ye ZL, Yang X (2009) Effect of triptolide on microinflammation in the patients with diabetic nephropathy. Journal of Clinical Nephrology, 9(2):82-84.

[23] Guo YQ, Zuo YH (2007) Clinical analysis of effect of Tripterysium glucosides in treatment of diabetic nephropathy. Journal of Clinical Nephrology 7:198-199.

[24] Fan HY, Shi YJ (2005) Effect of Triptolide on Transforming Growth Factor -β in Diabetic Nephropathy Cases. Chinese Journal of Integrated Traditional and Western Nephrology2005:395-397.

[25] Tu CF, Wang LJ, Gu LJ (2017) Effect of tripterygium wilfordii polyglycoside combined telmisartan on renal function and hemorheology in patients with diabetic nephropathy. Chinese Journal of General Practice15:1527-1528+1595.

[26] Jiang X (2015) Clinical observation of tripterygium glycosides combined with telmisartan in treatment 391 of diabetic nephropathy. Drugs & Clinic30:987-90. 392

[27] Wu YP, Shi NC (2018) Clinical observation on the effect of Valsartan combined with Tripterygium glycosides in the treatment of diabetic nephropathy stage Ⅳ. Chinese Remedies & Clinics 18:753-754.

[28]Xu GB, Chen DJ, Chen WZ (2017) Effect of Tripterygium Wilfordii Polyglycoside on Inflammatory FactorLevel in Patients with Diabetic Nephropathy, Chinese Archives of Traditional Chinese Medicine35:2206-2208.

[29] Zhang HC (2015) A clincial study of tripterygium glycosides tablets adjuvant treatment of diabetic nephropathy during Ⅳ period. China Pharmaceuticals 24: 248-249.

[30] Su HJ, Qian Y, Li HS (2013) Efficacy of tripterygium glycosides on proteinuria patients with diabetic nephropathy. Medical Journal of Wuhan University34:296-298.

[31] Ge YC, Xie HL, Li SJ (2010) Effect of tripterygium wilfordli in patients with diabetic nephropathy：a prospective randomized control clinical trial. Chinese Journal of Nephrology Dialysis & Transplantation 19:501-507+533.

[32] Wang ML,Zhang C (2012) Clinical observation of Valsartan combined with Tripterygium wilfordii in the treatment of proteinuria in type 2 diabetic nephropathy. Chinese Journal of Clinical Rational Drug Use 5: 84-85.

[33] Jiang AL, Chu XY, Wang HT (2014)Clinical observation on the effect of low-dose tripterygium glycosides on proteinuria in diabetic nephropathy. Chinese Journal of Integrated Traditional and Western Nephrology 15:444-445.

[34] Tang ZH, Luo DW, Yuan WJ (2017)A randomized controlled study of tripterygium glycosides polyglycoside in the treatment of diabetic nephropathy with moderate non-proliferative retinopathy. Chinese Journal of Integrated Traditional and Western Nephrology 18:332-334.

[35] Zhao RY, Tang BS, Shi XL et al (2011) Clinical observation on 46 cases of diabetic nephropathy treated withTtripterygium wilfordii and Valsartan. Chinese Journal of Integrated Traditional and Western Nephrology 12: 811-813.

[36] Yin ZH, Xia Li (2014) Clinical observation of tripterygium wilfordii polyglycoside on proteinuria in diabetic nephropathy. Lishizhen Medicine and Materia

[37] Zheng XP (2009) Clinical observation of tripterygium wilfordii on diabetic nephropathy. Journal of Clinical and Experimental Medicine. 8: 134-135.

[38] Ma RX, Zhao N, Zhang W (2013) The effects and m echanism of tripterygium wilfordii Hook F com bination with irbesartan on urinary podocyte excretion in diabetic nephropathy patients. Chin J Intern Med. 52: 469-73.

[39] Chang X, Li L, Wang B, et al (2020) Evaluation of the efficacy and safety of TWHF in diabetic nephropathy patients with overt proteinuria and normal eGFR. J Formos Med Assoc.119: 685-692.

[40] Ma RX, Xu Y, Jiang W, et al (2015) Combination of Tripterygium wilfordii Hook F and angiotensin receptor blocker synergistically reduces excretion of urinary podocytes in patients with type 2 diabetic kidney disease. Biotechnol Biotechnol Equip. 29: 139-146.

[41] Gao SP (2012) A randomized controlled study of Tripterygium wilfordii polyglycoside in the treatment of diabetic nephropathy. Hainan Medical Journal. 23: 31-32.

[42] Zheng YH (2014) Tripterygium wilfordii clinical research for the treatment of diabetic nephropathy stage IV[Master]: Guangzhou University of Chinese Medicine.

Figure 1.


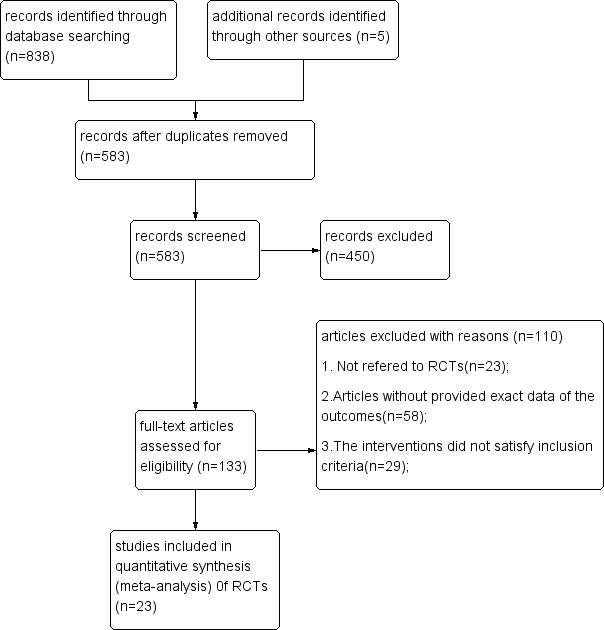


Figure 2.


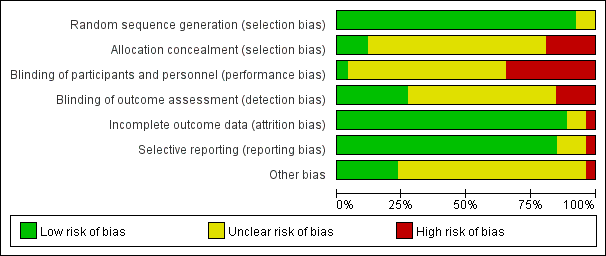


Figure 3.


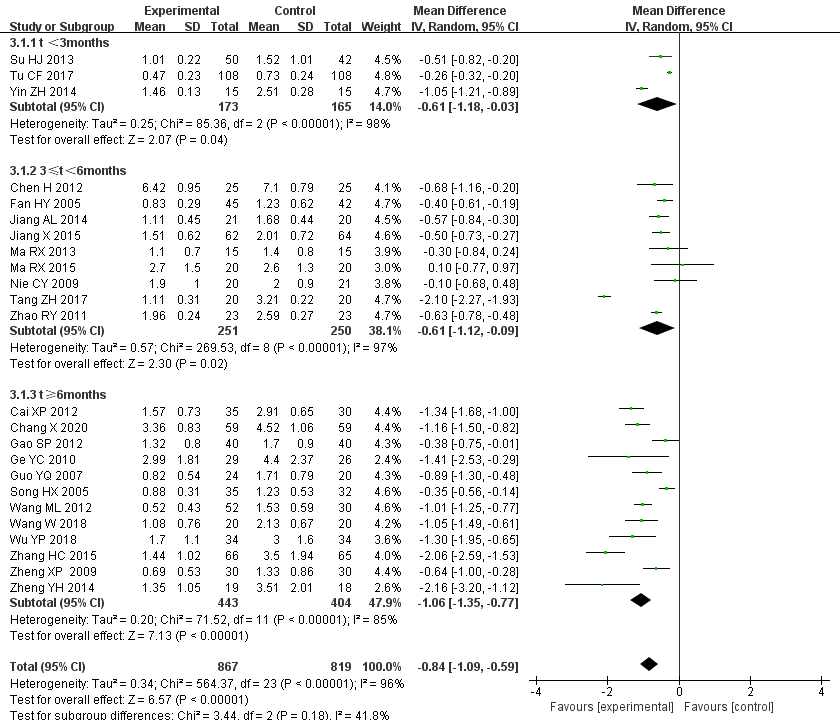


Figure 4.


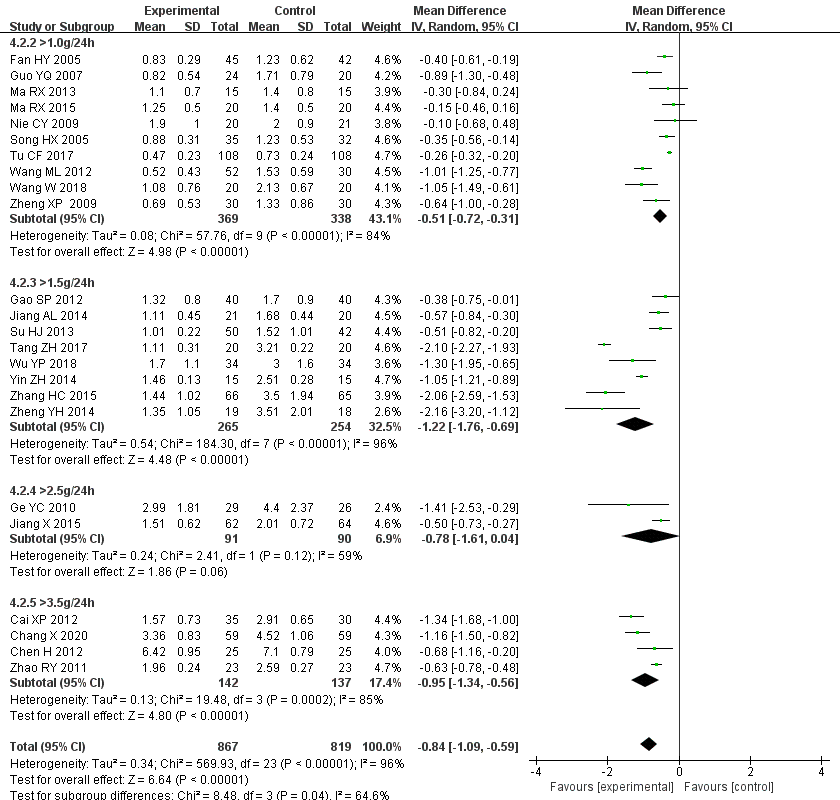


Figure 5


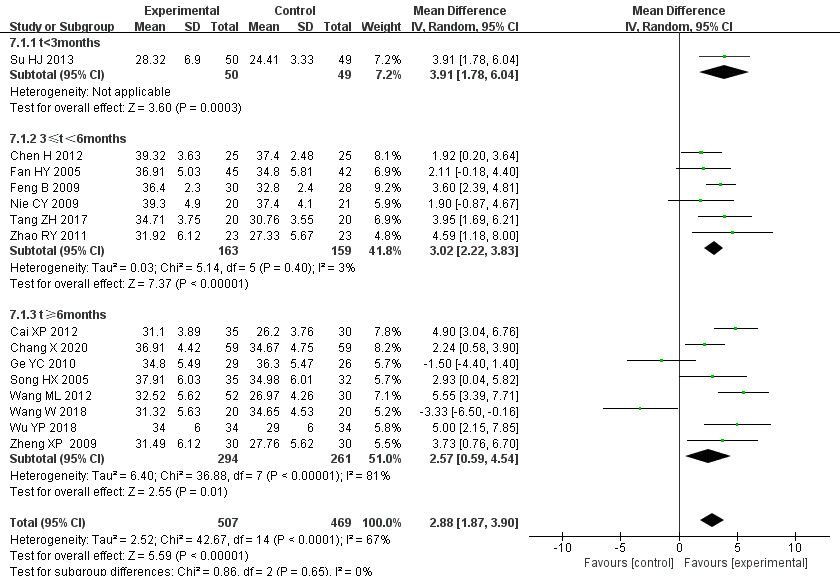


Figure 6.


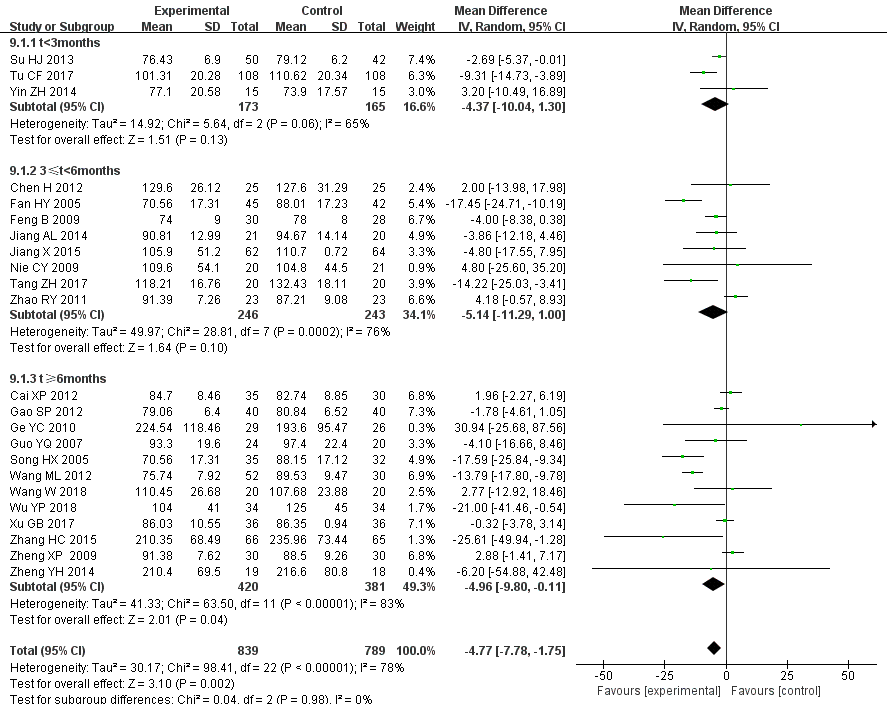


Figure 7


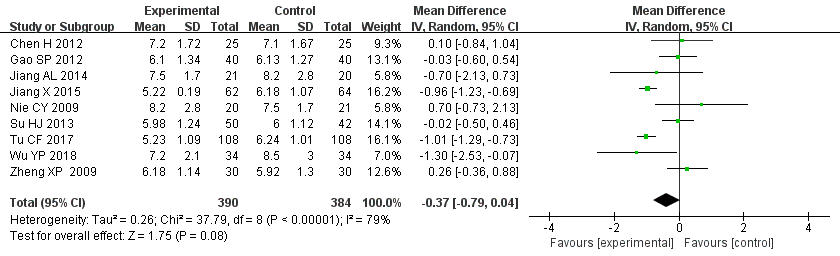


Figure 8


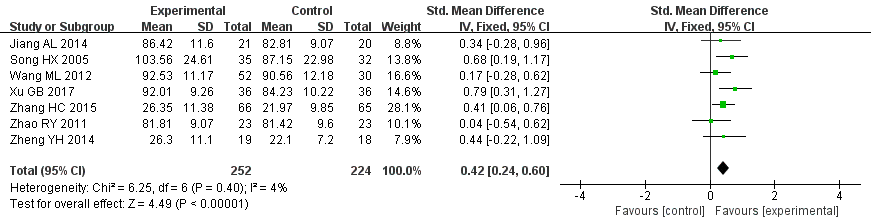


Figure 9.


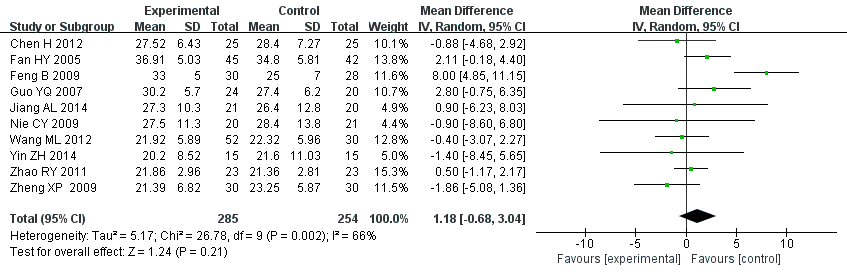


Figure 10.


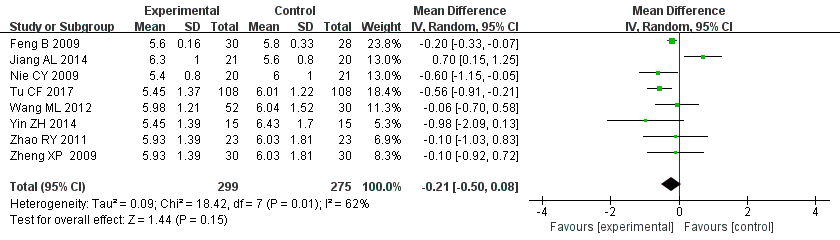


Figure 11.


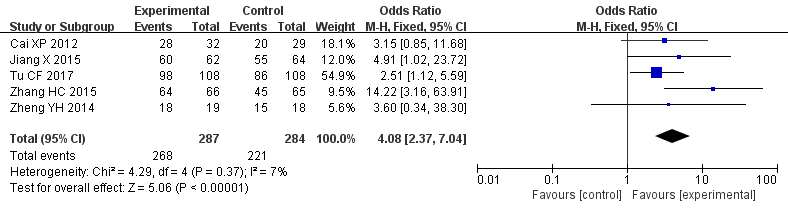

Supplement: Supplementary file 1 — Additional file 1: [file 12882_2021_2487_MOESM1_ESM.docx]
